# Greenhouse Gas Emissions and Health in the Countries of the European Union

**DOI:** 10.3389/fpubh.2021.756652

**Published:** 2021-12-10

**Authors:** Beata Gavurova, Martin Rigelsky, Viera Ivankova

**Affiliations:** ^1^Center for Applied Economic Research, Faculty of Management and Economics, Tomas Bata University in Zlín, Zlín, Czechia; ^2^Department of Marketing and International Trade, Faculty of Management, University of Prešov, Prešov, Slovakia; ^3^Institute of Earth Resources, Faculty of Mining, Ecology, Process Control and Geotechnologies, Technical University of Košice, Košice, Slovakia

**Keywords:** GHG, emissions, carbon dioxide, air quality, health, disability-adjusted life years, association, Europe

## Abstract

In the current era of globalization, a clean environment remains a crucial factor for the health of the population. Thus, improving air quality is a major focus of environmental policies, as it affects all aspects of nature, including humans. For these reasons, it is appropriate to take into account the health risks posed by greenhouse gas (GHG) emissions released into the atmosphere. With regard to global GHG emissions, there are concerns about the loss of protection of the ozone layer and it is very likely that climate change can be expected, which multiplies the environmental threat and has potentially serious global consequences. In this regard, it is important to pay increased attention to emissions that enter the atmosphere, which include countless toxic substances. The aim of this study was to examine the associations between selected GHG emissions and the health of the European Union (EU) population represented by disability-adjusted life years (DALYs). This aim was achieved using several analytical procedures (descriptive analysis, correlation analysis, cluster analysis, and panel regression analysis), which included five environmental variables (carbon dioxide (CO_2_), methane (CH_4_) in CO_2_ equivalent, nitrous oxide (N_2_O) in CO_2_ equivalent, hydrofluorocarbons (HFC) in CO_2_ equivalent, sulfur hexafluoride (SF_6_) in CO_2_ equivalent) and one health variable (DALYs). An emphasis was placed on the use of quantitative methods. The results showed that CO_2_ emissions have a dominant position among selected GHG emissions. The revealed positive link between CO_2_ and DALYs indicated that a decrease in CO_2_ may be associated with a decrease in DALYs, but it is also true that this cannot be done without reducing emissions of other combustion products. In terms of CO_2_, the least positive scores were observed in Luxembourg and Estonia. Germany had the lowest score of DALYs, representing the most positive health outcome in the EU. In terms of total GHG emissions, Ireland and Luxembourg were considered to be less positive countries compared to the other analyzed countries. Countries should focus on reducing GHG emissions in general, but from a health point of view, reducing CO_2_ emissions seems to be the most beneficial.

## Introduction

The world is dominated by ever-increasing production demands that leave traces in the environment. The other side of this coin is the degradation of the environment in connection with the production of greenhouse gas (GHG) emissions, climate change, loss of the protective ozone layer, global warming, which affect humanity, including its health (1, 2). According to a report published by the Intergovernmental Panel on Climate Change (3), humanity has been able to warm the planet over the past 50 years, while GHG emissions are considered a very serious aspect of human activity that contributes to this threatening state. These activities include the burning of fossil fuels, deforestation or intensive agriculture and others. Thus, it is important to pay increased attention to emissions that enter the atmosphere, which include countless toxic substances. In this sense, it is also possible to identify the world's biggest GHG emitters, and the fact remains that in 2015, the European Union (EU) ranked third behind China and the United States. The energy industry, agriculture, industrial processes, the use of products, and waste management were primarily responsible for this situation in the EU (4). These facts justify the importance of examining this problem in the EU. Reducing GHG emissions is a major environmental challenge of the 21st century and it is time to think about the current situation in different countries. An important message is the European Green Deal, a strategic plan to transform climate and environmental challenges into opportunities in order to build a modern, competitive and resource-efficient Europe. Striving to become the first climate-neutral continent also means a commitment to achieve zero net GHG emissions by 2050 (5).

It is the air that allows substances and chemicals to be transported around the world, while increasing air pollution and increasing GHG emissions almost always go hand in hand (6). Most GHGs have a long lifespan and are well-mixed in the ambient atmosphere. It is also true that emissions with the global warming potential can be harmful and degrade air quality, and their consequences can be reflected in global climate change affecting ecosystems and humanity (7, 8). It can be stated that GHG emission sources are all around. For instance, coal consumption in the energy sector is characterized by carbon dioxide (CO_2_) and sulfur dioxide (SO_2_) emissions. Rice cultivation, composting and livestock farming are dominant for methane (CH_4_) emissions, while the use of synthetic fertilizers in the agricultural sector is the main source of nitrous oxide (N_2_O) emissions (9–11). GHG and air pollutants share the same emission sources, and they come from the same natural and anthropogenic sources. In this regard, both air pollutants and CO_2_ are all products of combustion. Thus, part of the link between CO_2_ emissions and health should be explained by the air pollution that is emitted along with the CO_2_ (1). Despite the interconnection, air pollutant and GHG emissions do not need to be synchronized and require harmonization through well-developed strategies (9).

Focusing on GHG emissions, the greatest consequences of global climate change with an impact on human health are represented by increased temperatures, the longer duration of temperatures and their intensity. The heat also causes the occurrence of new pollen allergens and the number of premature deaths due to overheating of the body. In addition, more frequent forest fires caused by drought increase the number of cases with severe burns. All these facts indicate that there is no doubt that climate change is a current and future risk factor for health (2). In the context of the main ide of this study, it can be assumed that tackling emissions can mean tackling climate, global warming, the environment at the same time, while public health co-benefits can also be expected (1, 12, 13). It is well-known that health effects due to climate change are a result of global emissions, but a national perspective is also important and the following findings provide a rationale.

In fact, GHG emissions reduce the health potential of a population, which can be seen in the losses expressed by disability-adjusted life years (DALYs) (14). A study conducted by Woodcock et al. (15) supported the idea that reducing CO_2_ in London and Delhi through less use of motor vehicles and more use of lower-emission motor vehicles can lead to health benefits in terms of DALYs. Using a comparative method of health risk assessment in Malaysia, similar findings were revealed by Kwan et al. (16). This is in line with the fact that CO_2_ is a substance that is harmful to health and a key substance in climate change, thus its global warming potential may also be reflected in the DALYs indicator (17, 18). Thus, it can be concluded that population health (measured as DALYs) is damaged by factors related to carbon emissions (19, 20). Using a panel regression analysis, Dong et al. (21) also supported the idea that carbon emissions have a long-term adverse effect on population health, while a 1% increase in carbon emissions could lead to a 0.298% higher number of outpatients and a 0.162% higher number of inpatients. On this basis, it seems that efforts to reduce carbon emissions could be beneficial from both an environmental and a health point of view, as highlighted by findings in the research from Belgium, in which a carbon tax has proved to be a tool for preventing DALYs, as well as for saving healthcare spending (22). At the same time, not only reducing carbon emissions, but also reducing nitrous oxide and methane emissions provides an opportunity to improve the environment and public health as such (23, 24). Accordingly, GHG emissions such as CO_2_, CH_4_ and N_2_O are considered a health threat, even if the DALYs indicator is taken into account (25).

As pointed out, environmental aspects are related to a wide range of human lives, while GHG emissions are no exception. There is no doubt that GHG emissions could pose a greater or lesser threat to human health and the overall environment. Therefore, it is necessary to monitor emissions in individual countries, which simultaneously generate global emissions with a health effect in terms of climate change and global warming. International organizations emphasize the serious situation and call for active action to address it. The presented study provided a closer look at the links between GHG emissions and DALYs in the EU. The EU brings together countries that share the same goals, including health, but also economic and environmental ones (26, 27). In order to set effective strategies, it is important to know the situation in individual EU countries. The fact is that increased research attention has focused on climate change and its effects on public health, but no less important is the view of the link between GHG emissions and health. This issue has been overlooked and there is a lack of sufficient knowledge. The present study pointed to the trajectory of individual indicators and filled this gap in research, thus contributing to environmental and health issues from a unique point of view.

## Materials and Methods

### Research Objective

The primary aim of this study was to examine the associations between selected GHG emissions and the health of the EU population represented by DALYs.

The research sample consisted of 27 EU countries, with the oldest data were from 2009 and the most recent from 2018 (annual data without any missing values). The period was chosen based on the assumption of certain changes in mortality in connection with the economic crisis in 2008, as pointed out by Laliotis et al. (28). The following countries were included in the analytical process: Austria, Belgium, Bulgaria, Croatia, Cyprus, Czech Republic, Denmark, Estonia, Finland, France, Germany, Greece, Hungary, Ireland, Italy, Latvia, Lithuania, Luxembourg, Malta, Netherlands, Poland, Portugal, Romania, Slovakia, Slovenia, Spain, Sweden.

### Research Data

The data can be considered relevant, as their sources are databases of international organizations such as Eurostat, which obtain data from national statistical offices. The relevance of the Global Burden of Disease (GBD) Study database is also evidenced by the very close connection with the Lancet journal (29).

#### GHG Emissions

The analytical processes included five environmental indicators capturing GHG emissions, namely CO_2_, CH_4_ (in CO_2_ equivalent), N_2_O (in CO_2_ equivalent), HFC (in CO_2_ equivalent), and SF_6_ (in CO_2_ equivalent). The indicators representing GHG emissions were expressed in thousands of tons per 100,000 inhabitants. Data on GHG emissions as well as data on the average annual population in each country were collected from the Eurostat database (30) (Section: Environment and energy—Environment—Emissions of greenhouse gases and air pollutants—Air emissions accounts).

#### DALYs

The analyzes also included one indicator capturing health status, namely the DALYs indicator. This health indicator was expressed per 1,000 inhabitants of a particular country. The DALYs indicator expresses a measure of the gap in healthy years of life lived by a population as compared with a normative standard. In other words, DALYs are a time-based measure that adds together years of life lost due to premature mortality with the equivalent number of years of life lived with disability or illness. Thus, the DALYs indicator includes the years of life lost due to premature mortality (YLLs) and the years lived with a disability (YLDs) (31). Data on DALYs were obtained from the GBD database (32) [Section: IHME Data (TGBD Results Tool)], which can be considered as one of the largest databases for health-related indicators. Also, this indicator was expressed in terms of overall mortality (all diagnosis groups were included).

### Statistical Approach

Several methods and procedures were used in the statistical processing, which was divided into three consecutive subsections, namely (i) Univariate View—Statistical Description, (ii) Bivariate View of Associations—Correlation Analysis and Regression Analysis, and (iii) Multivariate View of Associations—Cluster Analysis.

The main analytical calculations were performed using the programming language R v 4.0.3 (RStudio, Inc., Boston, MA, US), while Tableau v 2020.2 (Tableau Software, LLC, Seattle, WA, U.S.) was used secondarily.

#### Analysis With a Univariate View

For the purpose of providing a statistical description, the basic statistical characteristics were used, namely mean, median, standard deviation, skewness, kurtosis, minimum, maximum, first quartile and third quartile. These results can be found in the first subsection Univariate View—Statistical Description.

#### Analyzes With a Bivariate View

Subsequently, in the second subsection Bivariate View of Associations—Correlation Analysis and Regression Analysis, the non-parametric coefficient (Spearman's ρ) was used in the correlation analysis to assess the associations. In this subsection, the assumptions for the selection of panel regression models were also evaluated. The Breusch-Pagan test (33) was applied to assess the variability of the residues. The use of Wooldridge's test for unobserved individual effects (34) was aimed at assessing the significance of unobserved effects through the distribution of residues, while in the case of significant effects, it was recommended to apply a regression model that takes into account the internal data structure. Due to a certain probability within which it was possible to assume the occurrence of a serial correlation, the Baltagi and Li one-sided LM test was chosen to assess this specificity (35). The F test for the presence of individual effects (or time effects) was used to assess the significance of effects in the internal data structure in terms of countries and individual years. The Hausman test and the robust regression-based Hausman test (vcov: vcovHC) were useful in choosing a model with fixed (within) effects or a model with random effects. The application of Angrist and Newey's test (36) supported the choice between the mentioned models in terms of identifying the limitations of models with fixed effects. Two variants of regression models were presented in a robust version, namely a model with fixed effects, i.e., Oneway (individual) effect Within model (Arellano estimator), and a model with random effects, i.e., Oneway (individual) effect Random effect model: Swamy-Arora's transformation (White 2 estimator). The result of the regression analysis points to the associations between GHG emissions (independent variables) and health represented by DALYs (dependent variable). The regression result has a higher added value compared to the correlation result. However, even this association cannot be considered causal, as the causal links were not proven by the analyzes used in this study. Compared to the classical model (Ordinary Least Squares—pooling model), the panel models take into account the internal data structure (in this case the structure within countries). The study provided the results of a pooling model and two panel models to compare estimates, but the preference for one of them and for its result was given by the above-mentioned assumptions.

#### Analysis With a Multivariate View

The statistical processing also included cluster analysis, the results of which are presented in the subsection Multivariate View of Associations—Cluster Analysis. The indices in this analysis were recalculated in several steps. In the first step, the arithmetic average of the individual indicators was calculated for each country (the results of this step are given in Supplementary Table 1). The average for countries included all observed years (in all countries the number of years was identical: 2009–2018; *n* = 10). Subsequently, these average values were standardized from 0 to 1 (0 represented the least positive value and 1 the most positive value). The standardization was based on an Equation (1):


(1) $$\begin{array}{l} z_{i}=1-\frac{x_{i}-\min\left(x\right)}{\max\left(x\right)-\min\left(x\right)} \end{array}$$

where *x*_*i*_ is the average of the reported values of the indicator for a specific country (for the observed period), *min(x)* is the lowest average value of the indicator (for the observed period) identified in the dataset containing all examined countries, *max(x)* is the highest average value of the indicator (for the observed period) identified in the dataset containing all examined countries.

In the subsequent step, the arithmetic average was calculated from the standardized values of individual GHG indicators for each country, thus creating a new variable—Greenhouse gases index. This index was developed for the purposes of this study and expresses a score ranging from 0 to 1, with 0 representing the least positive score in terms of all GHG emissions (a country with the highest average GHG production) and 1 representing the most positive score in terms of all GHG emissions (a country with the lowest average GHG production). The DALYs index was formed by only one variable, that is the standardized DALYs. This variable was created in the previous standardization step. The values adjusted in this way were the input to **Figure 2** and the cluster analysis (**Figure 3**). The cluster analysis included only two variables (Greenhouse gases index and DALYs index). The cluster analysis was performed using the Partitioning Around Medoids (PAM) algorithm (37) suitable for data in which the presence of outliers is expected, and using the algorithm of Manhattan distance. Simultaneously, a simpler cluster method, the k-means algorithm, was used. The optimal number of clusters was estimated using the silhouette method (38).

## Results

The results section provides three separate subsections according to the analysis and the purpose of the research. The first subsection, Univariate View—Statistical Description, offers a descriptive analysis, which was used for a more detailed presentation of selected indicators. The second subsection, Bivariate View of Associations—Correlation Analysis and Regression Analysis, presents the results of a correlation analysis that pointed to the associations between GHG emissions and DALYs in the EU. Several plots were also provided in this subsection. Furthermore, a regression analysis was also used to examine the significance of the associations between GHG emissions on DALYs, taking into account the internal structure of the data. In the third subsection, Multivariate View of Associations—Cluster Analysis, a cluster analysis identified homogenous clusters of EU countries in terms of assessing GHG emissions and DALYs. The other steps in this subsection were aimed at linking these environmental and health indicators in a more detailed specification of individual EU countries.

### Univariate View—Statistical Description

This subsection presents the selected indicators in a more detailed univariate view. Table 1 shows the basic descriptive measures, and it is clear that CO_2_ (mean = 655.56 thousands of tons) dominated among the examined GHG emissions in EU countries, while the lowest mean value was found for SF_6_ (mean = 0.82 thousands of tons). The values of skewness and kurtosis indicated some deviations from the normal distribution, but in most cases, there were small deviations. With a focus on the skewness measure, positive values were observed in all cases, indicating an increased presence of higher values and potential outliers in both GHG and DALYs indicators. A certain contamination of the normal distribution could also be detected by comparing the mean and median values. The values of minimum, maximum, first and third quartile also provided a closer look at the examined indicators. At this point, it should be noted that the dataset did not contain missing values and that all GHG emissions (except CO_2_) are given in CO_2_ equivalent.

**Table 1:** Descriptive analysis of GHG emissions (in thousands of tons per 100,000 inhabitants) and DALYs (in years per 1,000 inhabitants).

**Statistics**	**CO_**2**_**	**CH_**4**_**	**N_**2**_O**	**HFC**	**SF_**6**_**	**DALYs**
*n*	270	270	270	270	270	270
Mean	655.56	103.99	61.86	20.99	0.82	7.45
Median	605.22	96.8	52.57	17.04	0.51	2.58
Std. Dev.	357.4	43.51	32.05	12.89	1.01	11.01
Skewness	1.12	2.66	1.12	1.86	2.38	2.25
Kurtosis	2.9	9.44	0.53	4.62	5.13	4.46
Minimum	−35.27	36.66	8.92	4.55	0.02	0.24
Maximum	2186.22	304.96	151.18	84.93	4.67	49.53
Quartile I	423.24	81.89	39.71	11.83	0.22	1.37
Quartile III	877.62	116.27	69.71	26.48	0.86	9.31

*n, frequency of observations; Std. Dev., standard deviation; Quartile I, first quartile (25th percentile); Quartile III, third quartile (75th percentile)*.

In general, it can be stated that not only CO_2_ but also CH_4_ requires attention with the second highest mean value among all emissions. An increased attention should also be paid to emissions such as N_2_O and HFC. On the contrary, the lowest mean value (in thousands of tons per 100,000 inhabitants) was found in SF_6_. The production of each of these gases is relatively specific. For example, CO_2_ is produced mainly by the combustion of fossil fuels, which seems to be easier to manage than N_2_O, which is produced predominantly in agriculture. This idea underlines the fact that, in addition to assessing the global warming potential, the potential for reducing specific GHG emissions should be taken into account.

When focusing on CO_2_, some deviations from other GHG emissions were evident. The level (in thousands of tons per 100,000 inhabitants) of other GHG emissions was much lower than the level of CO_2_ (e.g., SF_6_). Thus, the significant amount of CO_2_ emissions means that there is a lot of space to reduce its production. There were several countries showing clear evidence that this is possible (Supplementary Table 1 provides values for comparison between countries). The sample included countries where CO_2_ emissions ranged from very high level (Luxembourg) to very low level (Sweden). When assessing GHG emissions, it is also necessary to take into account their negative effects, which are not proportional, as some GHGs can affect global warming more than others. Some gases occur less in the atmosphere, but they capture heat several times more effectively than CO_2_. In this study, the global warming potential of individual greenhouse gases was expressed in terms of CO_2_, as the CO_2_ equivalent was used for other emissions. The analysis also included one health indicator, based on the results of which it can be stated that the EU population lived on average 7.45 healthy life years less than they could (calculated per 1,000 inhabitants).

### Bivariate View of Associations—Correlation Analysis and Regression Analysis

This subsection points out the associations between the analyzed indicators in a bivariate view.

**Figure 1:**
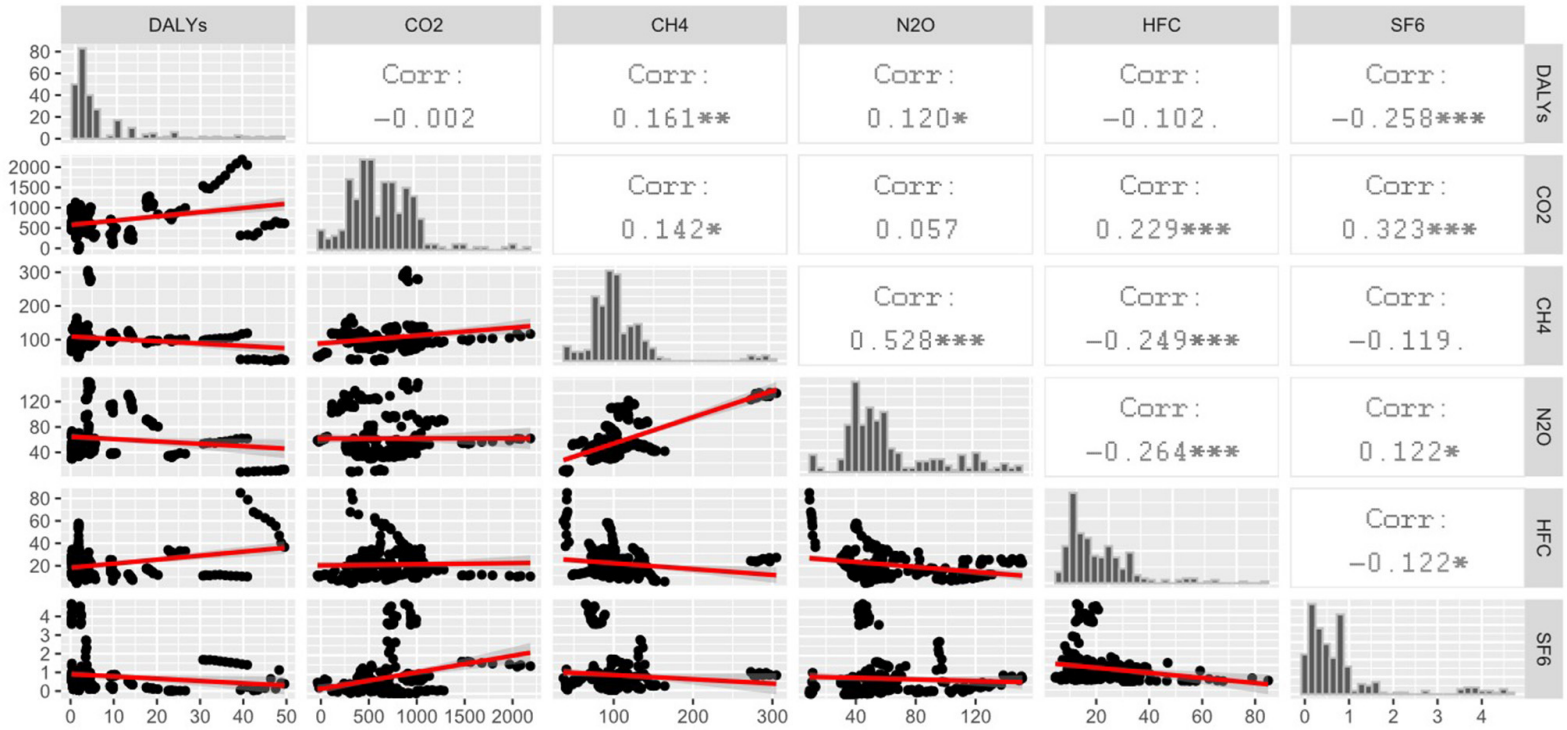
Correlation analysis of GHG indicators (in thousands of tons per 100,000 inhabitants) and DALYs (in years per 1,000 inhabitants) without classification of EU countries, and plots of individual indicators. Sig: 0.1; *0.05; **0.01; ***0.001.

Figure 1 completes the information on GHG and DALYs indicators. Thus, it clarifies their structure as well as the associations between them in order to better understand the issue. Density plots are shown in a diagonal line, scatter plots are below the diagonal line, and correlation coefficients are above the diagonal line (Spearman's ρ). Regarding the associations between individual GHG emissions and DALYs, a significant correlation was found for CH_4_, N_2_O and SF_6_. In these cases, a significance could be confirmed at the level of α < 0.05. However, it is necessary to distinguish between positive and negative correlation coefficients. A negative correlation was found between SF_6_ and DALYs (ρ = −0.258). It is also useful to focus on the associations between the individual GHG indicators, within which it was possible to find the highest rate of correlation between N_2_O and CH_4_ (ρ = 0.528). Furthermore, it was possible to point out a positive correlation between SF_6_ and CO_2_ (ρ = 0.323), as well as a negative correlation between HCF and N_2_O (ρ = −0.264).

Table 2 shows the correlations between GHG emissions and DALYs in terms of individual EU countries. In other words, the table shows the associations in which the inputs were individual GHG emissions and DALYs for each country in individual years (*N* = 10). A significant positive correlation coefficient indicated that in the years when a country showed a higher value of DALYs, it was also possible to observe a higher value of a certain GHG emission and vice versa. On the other hand, a significant negative coefficient indicated that in the years when a higher value of DALYs was measured, it was possible to observe a lower value of a certain GHG emission and vice versa. Across EU countries, significant positive correlations were found mainly for CO_2_ and CH_4_, while the highest number of negative correlations was found for HCF. Supplementary Table 1 provides the average values of the examined indicators in individual EU countries, as well as the inclusion of countries in the cluster.

**Table 2:** Correlation coefficients between GHG emissions and DALYs by EU country.

**ID**	**CO_**2**_**	**CH_**4**_**	**N_**2**_O**	**HFC**	**SF_**6**_**
AUT	0.750^**^	0.995^†^	0.611^*^	−0.935^†^	−0.486
BEL	0.894^†^	0.988^†^	0.919^†^	−0.966^†^	0.479
BGR	−0.375	0.142	−0.388	−0.175	−0.640^*^
CYP	0.811^***^	0.177	0.777^***^	0.094	0.017
CZE	0.562^*^	0.893^†^	−0.711^**^	−0.984^†^	0.839^***^
DEU	0.811^***^	0.967^†^	0.499	−0.183	−0.923^†^
DNK	0.881^†^	0.980^†^	0.556^*^	0.974^†^	−0.453
ESP	0.834^***^	0.800^***^	−0.164	0.723^**^	0.496
EST	−0.657^**^	0.768^***^	−0.935^†^	−0.975^†^	−0.970^†^
FIN	0.117	0.980^†^	0.809^***^	0.786^***^	−0.017
FRA	0.922^†^	0.991^†^	0.778^***^	0.069	0.956^†^
GRC	0.278	0.106	0.412	−0.425	0.170
HRV	0.564^*^	0.006	0.769^***^	−0.933^†^	0.662^**^
HUN	0.552^*^	0.801^***^	−0.905^†^	−0.640^**^	−0.651^**^
IRL	0.748^**^	−0.875^†^	−0.509	−0.630^*^	0.061
ITA	0.945^†^	0.947^†^	0.944^†^	−0.958^†^	0.425
LTU	−0.863^***^	0.710^**^	−0.506	−0.773^***^	−0.498
LUX	0.952^†^	0.953^†^	0.978^†^	−0.709^**^	−0.987^†^
LVA	−0.248	−0.895^†^	−0.937^†^	−0.962^†^	−0.925^†^
MLT	0.934^†^	−0.191	0.929^†^	−0.974^†^	0.590^*^
NDL	0.820^***^	0.959^†^	0.661^**^	0.906^†^	0.569^*^
POL	−0.238	0.903^†^	−0.545	−0.148	−0.849^***^
POR	−0.393	0.604^*^	0.255	−0.939^†^	0.881^†^
ROU	0.648^**^	0.812^***^	−0.256	−0.663^**^	−0.237^***^
SVK	0.576^*^	0.689^**^	0.752^**^	−0.895^†^	0.780^***^
SVN	−0.758^**^	0.958^†^	0.539	−0.861^***^	0.535
SWE	0.893^†^	0.992^†^	0.815^***^	0.879^†^	0.808^***^

*Sig*:

* *0.1*;

** *0.05*;

*** *0.01*;

† *0.001. Negative correlations are highlighted in red and positive correlations are highlighted in green. A richer color indicates a stronger correlation. Non-significant correlations are not highlighted*.

*ID, country identifier; AUT, Austria; BEL, Belgium; BGR, Bulgaria; CYP, Cyprus; CZE, Czech Republic; DEU, Germany; DNK, Denmark; ESP, Spain; EST, Estonia; FIN, Finland; FRA, France; GRC, Greece; HRV, Croatia; HUN, Hungary; IRL, Ireland; ITA, Italy; LTU, Lithuania; LUX, Luxembourg; LVA, Latvia; MLT, Malta; NDL, Netherlands; POL, Poland; POR, Portugal; ROU, Romania; SVK, Slovakia; SVN, Slovenia; SWE, Sweden*.

Table 3 provides an evaluation of the assumptions for selecting appropriate regression models. The results of the test to verify the constancy of residue variability (Breusch Pagan) indicated the presence of significant heteroscedasticity in almost all cases, while this contamination was not confirmed only for N_2_O. The serial correlation was verified using Wooldridge's test for unobserved individual effects and the Baltagi-Li one-sided LM test. Based on the results, it was possible to confirm the significance in all cases at the level of α < 0.05. Therefore, the choice of robust estimation methods was considered appropriate. The results of the F test applied within the data structure from the point of view of countries showed a significance in all of the analyzed cases at the level of α < 0.001. On the contrary, the results of the F test applied within the data structure from the point of view of years were not significant in any of the analyzed cases. Based on these results, only the effect resulting from the country structure was taken into account in the regression models. The preference of a fixed effects model or a random effects model was supported by the results of the tests in the last three rows of the table. If the results of the Hausman test and its robust version [Hausman (vcovHC)] showed a significance, a fixed effects model was preferred, otherwise a random effects model was considered more appropriate. In general, the result of Angrist and Newey's test with a *p*-value of < 0.05 indicates significant limitations of the model with fixed effects. In Table 3, it was possible to observe certain limitations in the model containing CO_2_ as an independent variable. Based on this fact, it was appropriate to take into account the results of both models (random and fixed) when assessing the association between CO_2_ emissions and DALYs. Apart from this exception, the recommendations for model preference in the other analyzed cases were clear.

**Table 3:** Assumptions of regression models with GHG emissions as independent variables and DALYs as a dependent variable.

**Assumptions**	**CO_**2**_**	**CH_**4**_**	**N_**2**_O**	**HFC**	**SF_**6**_**
Breusch Pagan	98.24^†^	5.77^**^	1.18	73.14^†^	13.89^†^
Wooldridge	3.06^***^	1.47	2.82^***^	2.68^***^	1.88^*^
Baltagi Li LM	9.19^†^	12.94^†^	11.79^†^	13.31^†^	11.29^†^
F Test Country	214.73^†^	842.65^†^	592.10^†^	70.59^†^	222.84^†^
F Test Year	0.18	0.11	0.01	0.79	0.01
Hausman	33.00^†^	4.91^**^	1.79	59.95^†^	0.96
Hausman (vcovHC)	33.11^†^	22.49^†^	2.12	2.29	0.38
Angrist and Newey	115.95^**^	85.65	86.96	68.47	31.88
Model	R/F	F	R	R	R

*Sig*:

* *0.1*;

** *0.05*;

*** *0.01*;

† *0.001; R, random effects model; F, fixed (within) effects model*.

Table 4 shows the results of the regression models. Despite the not entirely clear recommendation of the model preference for CO_2_, it was possible to observe a significant positive coefficient in both models (fixed and random). A significant association was also found for N_2_O, which also showed a positive β coefficient. Accordingly, a decrease in CO_2_ and N_2_O emissions was associated with a decrease in DALYs in EU countries and vice versa. In the case of CH_4_, the fixed (within) effects model was recommended, the result of which did not prove to be significant. The significant associations with DALYs could not be confirmed even in the analyzed cases of HFC or SF_6_. The significance of the associations between GHG emissions and DALYs was assessed on the basis of β coefficients, but the determination coefficients (*R*^2^) also provided useful information in assessing the associations. The highest values were found for CO_2_.

**Table 4:** Regression analysis results: associations between GHG emissions (independent variables) and DALYs (dependent variable).

**DALYs—dependent variable**		**CO_**2**_**	**CH_**4**_**	**N_**2**_O**	**HFC**	**SF_**6**_**
Pooling	β (SE)	10.41 (9.97)	−0.70 (0.55)	−0.38 (0.50)	0.36 (0.34)	−0.01 (0.01)
	β (SE)	578.04^†^ (70.9)	109.17^†^ (10.13)	64.68^†^ (6.77)	18.34^†^ (2.58)	0.91^†^ (0.25)
	*R* ^2^	0.1	0.03	0.02	0.09	0.01
Within	β (SE)	49.71^†^ (12.51)	1.05 (0.67)	0.46 (0.28)	−2.16 (1.35)	0.01 (0.03)
	*R* ^2^	0.32	0.05	0.01	0.2	0.002
Random	β (SE)	35.07^†^ (6.19)	0.82^***^ (0.26)	0.31^**^ (0.15)	−0.52 (0.38)	0.002 (0.01)
	α (SE)	394.35^†^ (71.99)	97.85^†^ (8.76)	59.56^†^ (6.40)	24.84^†^ (2.94)	0.81^†^ (0.22)
	*R* ^2^	0.24	0.03	0.006	0.03	<0.001

*Sig*:

* *0.1*;

** *0.05*;

*** *0.01*;

† *0.001*.

### Multivariate View of Associations—Cluster Analysis

**Figure 2:**
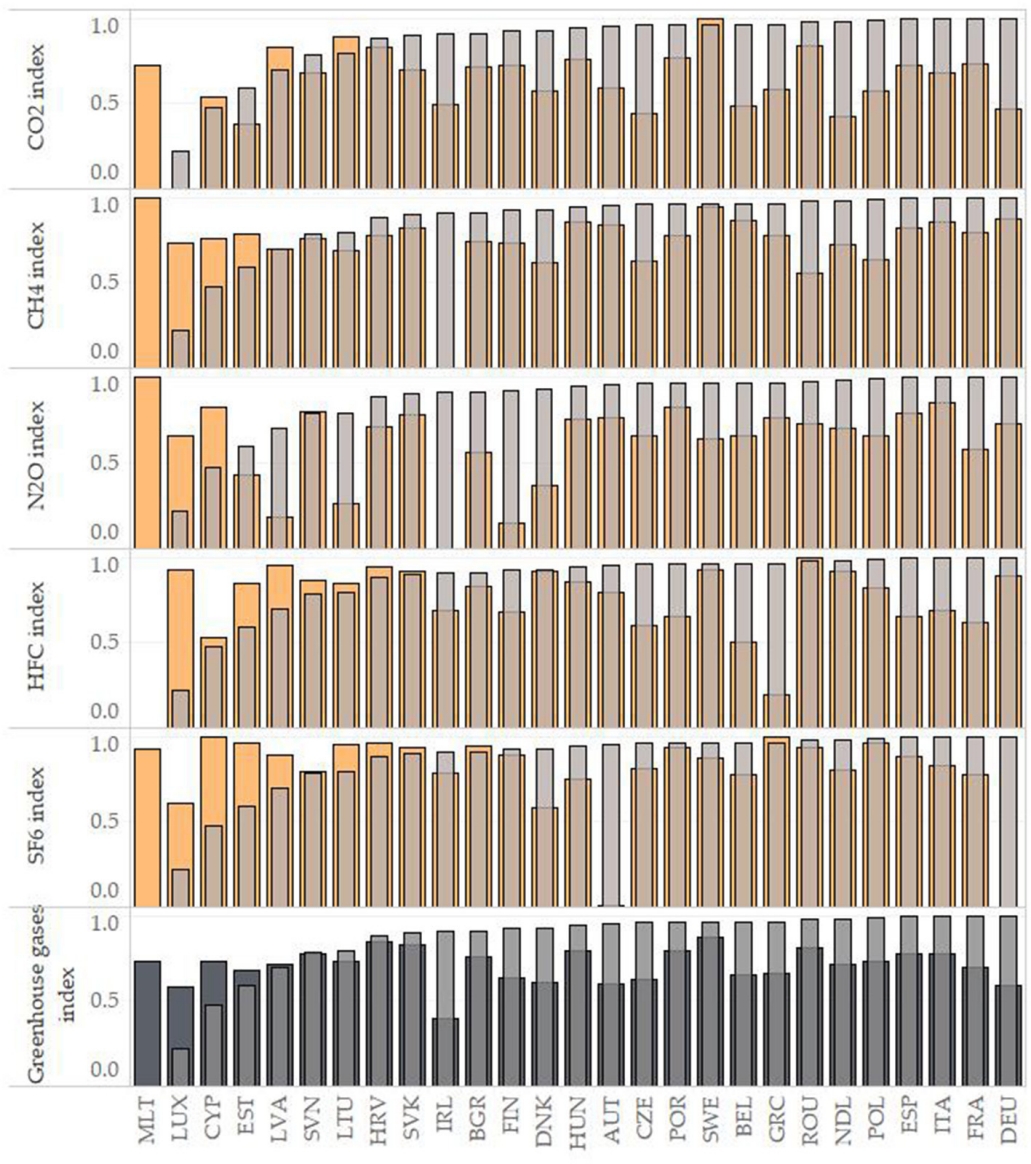
Scores of DALYs index and GHG indices in individual EU countries. Note: The lower the column the less positive the assessment. The countries are ranked from the lowest to the highest DALYs index. The DALY index is shown in each partial graph by a transparent gray color, the individual GHG indices by a yellow color and the Greenhouse gases index by a dark opaque gray. AUT, Austria; BEL, Belgium; BGR, Bulgaria; CYP, Cyprus; CZE, Czech Republic; DEU, Germany; DNK, Denmark; ESP, Spain; EST, Estonia; FIN, Finland; FRA, France; GRC, Greece; HRV, Croatia; HUN, Hungary; IRL, Ireland; ITA, Italy; LTU, Lithuania; LUX, Luxembourg; LVA, Latvia; MLT, Malta; NDL, Netherlands; POL, Poland; POR, Portugal; ROU, Romania; SVK, Slovakia; SVN, Slovenia; SWE, Sweden.

This subsection points to the associations between the analyzed indicators in a multivariate view. Figure 2 shows the GHG emission indices and the DALYs index in individual EU countries. The indices ranged from 0 to 1 (standardized score), where 0 was the least positive score and 1 was the most positive score in terms of GHG emissions as well as DALYs. The calculation of GHG indices (CO_2_ index, CH_4_ index, N_2_O index, HCF index, SF_6_ index, DALYs index) was performed using the arithmetic average of the values of individual indicators for the observed period in each EU country and subsequent standardization. The Greenhouse gases index represented the average of individual indices of GHG emissions.

Based on the results in Figure 2, Malta (MLT) could be considered as an outlying country due to the acquired slightly asymmetric values. This country showed the least positive score of the DALYs index but, in several cases, a very positive score of the GHG emission indices. Among the EU countries, Germany showed the most positive score of the DALYs index, followed by France, Italy and Spain. In terms of total GHG emissions in countries (Greenhouse gases index), the least positive score was identified in Ireland, followed by countries such as Luxembourg, Germany and Austria. On the other hand, countries such as Sweden, Croatia and Slovakia could be considered positive. With a focus on the indicator with a dominant position among selected GHG emissions, CO_2_, the least positive scores were found in Luxembourg and Estonia. Based on the results of the regression analysis, these countries have great opportunities to improve the environment as well as the health of their population.

**Figure 3:**
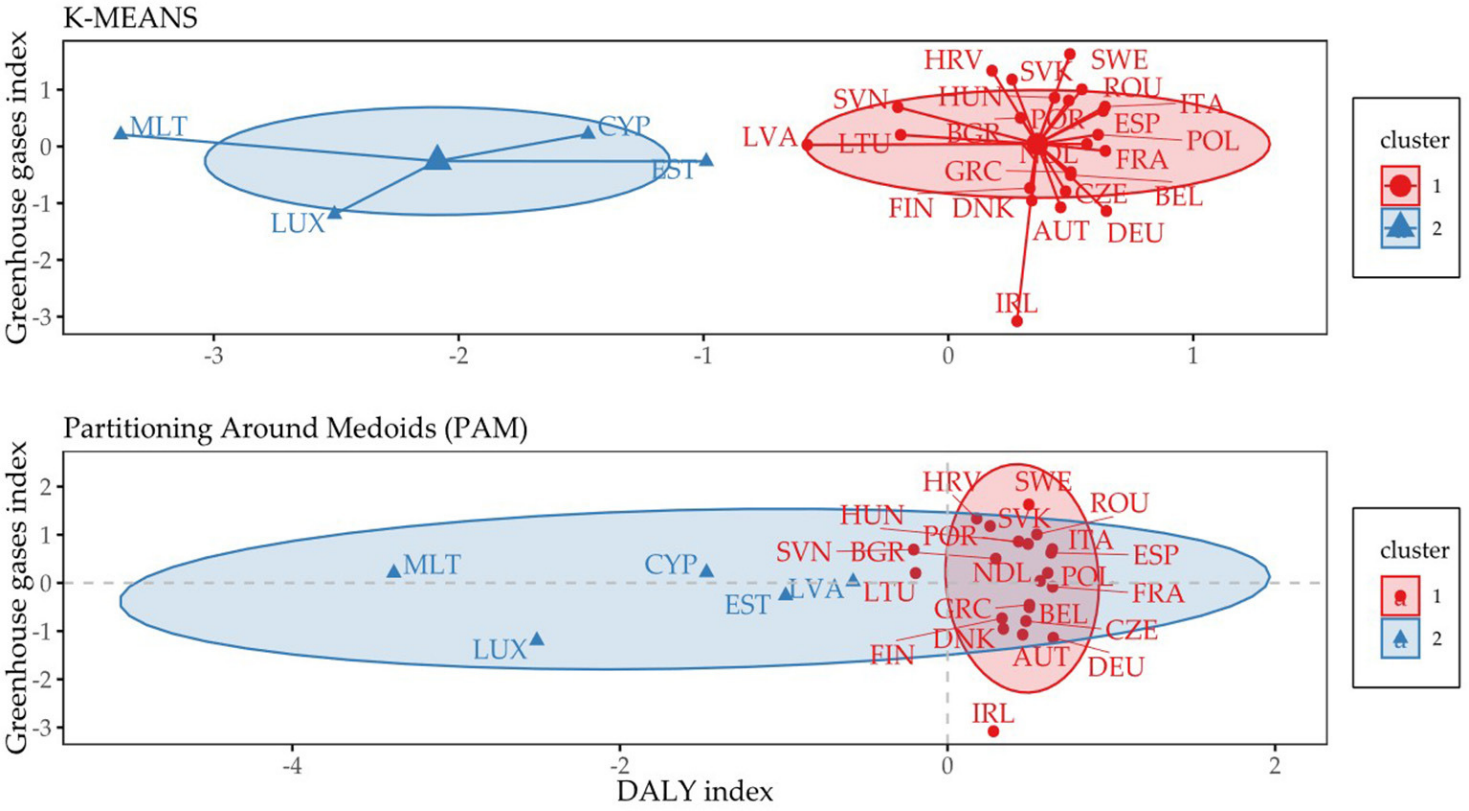
Cluster maps: Country positions based on the DALYs index score and the Greenhouse gases index score. AUT, Austria; BEL, Belgium; BGR, Bulgaria; CYP, Cyprus; CZE, Czech Republic; DEU, Germany; DNK, Denmark; ESP, Spain; EST, Estonia; FIN, Finland; FRA, France; GRC, Greece; HRV, Croatia; HUN, Hungary; IRL, Ireland; ITA, Italy; LTU, Lithuania; LUX, Luxembourg; LVA, Latvia; MLT, Malta; NDL, Netherlands; POL, Poland; POR, Portugal; ROU, Romania; SVK, Slovakia; SVN, Slovenia; SWE, Sweden.

Figure 3 provides maps of individual countries included in the created clusters based on the linking of the acquired DALYs index and the Greenhouse gases index (based on Equation 1). Two clustering methods were used, the k-means algorithm, the output of which is shown in the upper graph, and the PAM algorithm, the output of which is shown in the lower graph. Malta, Luxembourg, Cyprus, Estonia and possibly Latvia (cluster 2) could be considered as countries with different values obtained in the assessed dimensions compared to the countries in the first cluster. The PAM map can be interpreted in such a way that the closer the country is to the right side of the graph (x-axis), the more positive the DALYs index obtained. Simultaneously, the higher the country (y-axis), the more positive the score in total emissions (Greenhouse gases index). Thus, the link can be seen in such a way that the closer the country is to the upper right corner, the more positive the country's position in terms of the DALYs index and the Greenhouse gases index. Accordingly, Sweden was the country with the most positive assessment.

The k-means map presented in Figure 3 shows ellipses that were formed based on the Euclidean distance, and the PAM map shows ellipses that were formed using the distance based on the distribution of t. In general, it can be stated that a country outside an ellipse in the PAM map is a country with different scores compared to other countries in an ellipse. On this basis, it was possible to assess Ireland in a negative sense in terms of GHG emissions.

## Discussion

Humanity does not realize that GHG have existed in the atmosphere for a very long time. It is possible to speak of several years, decades or several thousand years. These gases affect the entire planet, regardless of which country produced them. Several studies declare an explicit link between health and environmental degradation, while evidence suggests that many diseases as well as premature deaths can be attributed to poor air quality and GHG emissions (12, 13, 39). In this already serious situation, it is desirable for each country to behave responsibly and to adopt emission reduction programs (40, 41). These facts were the motivation for examining the associations between GHG emissions and the health of the EU population represented by DALYs. First of all, the results identified the true state of GHG emissions in the EU, and a more detailed insight was provided using the descriptive analysis. The highest mean value was observed for CO_2_ and the lowest mean value was observed for SF_6_. This study supported the well-known fact that CO_2_ is considered to be one of the largest contributors to poor atmospheric quality among GHG emissions, and this finding was also confirmed by other authors (7, 17, 18, 25). It should be noted that CO_2_ accounts for up to 82% of world emissions, while CH_4_ accounts for 11%, N_2_O for 5%, HFC for 2% and SF_6_ for <0.2% (4). For DALYs in the EU, the results indicated a mean value of 7.45, which can be seen as an average of 7.45 years of life lost due to premature mortality, together with years lived with disability or illness. On the other hand, a median value was 2.58 years, which indicated deviations in EU countries.

In terms of comparing the observed DALYs and GHG emissions in individual countries, Germany was considered to be a country with the most positive score of DALYs, although this country acquired the third least positive score of total GHG emissions. On the other hand, Malta was considered to be a country with the least positive score of DALYs, but this country was characterized by the most positive score of emissions. These findings are consistent with other evidence (4), and the authors of this study call for further research to clarify which factor better explains these results. The least positive score of total GHG emissions was found in Ireland, while this country also showed a less positive score of DALYs from all EU countries. Only nine countries achieved worse DALYs than Ireland.

The associations between GHG emissions and DALYs within the EU as a whole were assessed using Spearman's ρ, and the significant correlations were confirmed for CH_4_, N_2_O a SF_6_. In individual EU countries, significant positive correlations with DALYs were found mainly for CO_2_ and CH_4_, while the highest number of negative correlations was found for HCF. In the regression analysis, the significant and positive associations with DALYs were found for both CO_2_ and N_2_O. This finding indicates that a decrease in CO_2_ and N_2_O emissions may be associated with a reduction in DALYs, which can be considered desirable from an environmental and health point of view. In this way, it is possible to increase the level of population health, however, it should be noted that this cannot be done without reducing emissions of other combustion products, which were not the subject of this study (1). This finding can be explained by their global warming potential and climate change leading to heat stress (2). The significant associations with DALYs were not confirmed for CH_4_, HFC or SF_6_. This finding does not necessarily mean that CH_4_, HFC and SF_6_ are not associated with health, as these emissions also contribute significantly to climate change, ozone depletion and global warming.

The findings of this study are in line with the findings of several authors who identify CO_2_ emissions as a health damaging factor in general (21), but also in terms of DALYs (17, 19, 20). Thus, it is possible to agree that CO_2_ emissions are significantly associated with public health (21) which emphasizes the need to implement effective measures to improve health, but also to improve the environment. One such measure seems to be a carbon tax as an effective tool for achieving health benefits in terms of DALYs as well as economic benefits (22). Another opportunity could be to increase the price of emission allowances and reduce the maximum number of emission allowances. In addition, there is no doubt that measures to reduce the use of motor vehicles in cities also play an important role, as this step can lead to a reduction in DALYs (15, 16). However, as pointed out in this study, not only CO_2_ is associated with health, the results showed a significant association between N_2_O and DALYs, which is in line with the idea of Montagu et al. (23), who saw low emissions of nitrous oxides as a win for health and the environment. It should also be noted that although this research did not support another association, other authors identified a reduction in CH_4_ emissions as an opportunity to improve air quality and public health as such (24). For these reasons, environmental policy and decision-making should apply a comprehensive approach to GHG emissions (42–44), as public health co-benefits can also be expected when reducing them (1, 12, 13).

Based on the findings revealed by the cluster analysis in combination with the results in Supplementary Table 1, countries such as Malta, Luxembourg, Cyprus, Estonia and possibly Latvia were considered to be lagging behind other EU countries, especially in terms of DALYs. These countries have great potential for improving health. Special attention should also be paid to Ireland, where the least positive score of total GHG emissions was observed. As the significant association between CO_2_ and DALYs was identified, countries with the least positive CO_2_ score also have great potential for improving health. By reducing CO_2_ emissions, countries such as Luxembourg, Estonia, but also Ireland can expect health benefits in terms of DALYs. On the other hand, this cannot be done without reducing emissions of other combustion products (1).

### Policy Implications

The results of our analyzes represent valuable findings for health and environmental policy makers, as well as support the creation of a platform for the development of strategic plans focused on reducing GHG emissions in the future. The health effects due to climate change are a result of global emissions, but the global success of reducing emissions cannot be achieved without active action in individual countries. However, it is first necessary to know the situation in countries, and this view in the EU was offered by this study. It is organizations such as the EU or the Organization for Economic Co-operation and Development (OECD) that require emissions control in their Member States, and it is therefore essential that policy-makers have up-to-date assessments of environmental emissions and public health in countries (14). None of the ambitious strategic objectives of reducing emission at international level can be achieved without evidence-based actions performed in individual countries. In any case, the results of the analyzes provided by this study are a good basis for successful measures. Policy makers have a unique position in the adoption and support of evidence-based measures with an effort to improve the health of the population, as well as the environment. Cooperation between researchers and policy makers in the field of environment and health could have great potential (45). Green and ecological mechanisms to reduce carbon footprint and improve energy efficiency should be developed in many dimensions of the country's life (46, 47). It would also be an effective way to take action at the population level, while programs aimed at educating people about protecting the environment and reducing emission from the consumer world play an important role. In many countries, environmental health is currently becoming a research and political domain rather than educational. This also justifies the importance of improving environmental literacy, which is considerably underestimated in many countries. Environmental literacy is that it comprises an awareness of and concern about the environment and its associated problems, as well as the knowledge, skills, and motivations to work toward solutions of current problems and the prevention of new ones (48).

### Strengths

The study enriches the current state of knowledge by clarifying the associations between GHG emissions and public health represented by DALYs. The analyzes respected the internal structure of the data. An emphasis was placed on the use of quantitative methods. The results of this study offer a fundamental and much-needed pillar for further research on this issue at the national and international levels. The study also showed the real situation in individual EU countries and provided a comparison of these countries. The findings are of great importance for the development of evidence-based plans in terms of improving the environment and the health of the population.

### Limitations

Potential limitations may include the endogeneity problem that may occur in regression analysis. Given this potential limitation, the results should be accepted with some caution. Another potential limitation can include the fact that the GHG indices entering the analytical procedures were not adjusted (they were not weighted) in any way, either according to the intensity of occurrence, or to their health effects. A certain limitation is also the fact that the health indicator (DALYs) presents the outcome for the total population (regardless of age, gender, etc.) and all diagnoses. With a focus on the correlation analysis, it is necessary to be careful in interpretations, as the revealed correlations were supported on a very small sample. Regarding the limitations of the regression model, it should be noted that the model used in this study did not examine causality as such, and therefore the results cannot be interpreted as causal links. All results can only be seen in terms of associations, while a consideration of causal links can be misleading. In addition, the model did not include control variables for better investigation of the revealed associations. Last but not least, the specific nature of environmental and health policies in countries needs to be emphasized. Each country takes a different approach to reducing GHG emissions, and ignoring this fact can be seen as a limitation. However, by using techniques respecting the specificity of the data structure (panel regression analysis), this shortcoming could be adequately recorded, captured and incorporated into computational processes. A similar limitation can be identified in the health indicator. There may be differences in the prevalence of individual diseases between countries. A certain disease may occur in one country more than in another. However, the mechanism for calculating the DALYs indicator reduced these differences. Also, the application of panel models captured possible specifics of countries and incorporated them into computational processes. In the case of this study, it can be assumed that the difference in GHG emissions and DALYs due to the specifics of countries did not skew the main results.

### Future Directions

From a computational perspective, future research activities should focus mainly on assessing the causal links between environmental indicators and DALYs. In the future, it will also be appropriate to include economic variables that allow for the approximation of countries' industries and a more accurate identification of emission sources. From this perspective, future research ambitions could also focus on the spatial panel regression method. Also, the DALYs indicator could be decomposed according to age groups, gender or specific diagnoses. An ambition for future research could also be the expansion of a sample of countries, e.g., involvement of other continents or other international communities, such as OECD countries.

## Conclusion

The study focuses on the proclaimed issue of the environment and human health in EU countries. The primary aim of this study was to examine the associations between selected GHG emissions and the health of the EU population represented by DALYs. Based on the results, it was possible to conclude that there is a certain link between GHG emissions and DALYs, while a dominant result was identified for CO_2_, which can be considered as a health risk factor. The study also calls for a more comprehensive analysis of the associations between emissions and population health and emphasizes the need for a multidisciplinary examination of the issue. The results of the study showed valuable findings that are important for EU countries and their leaders. In fact, the exact determination of the effects of individual GHG emissions on the deterioration of health is also problematic. This problem is also based on the global perspective of emissions released into the atmosphere. Therefore, the results of this study should not be seen as a clear link between national GHG emissions and national DALYs, and the global effects of emissions and climate change should be taken into account in public policy-making. Nevertheless, the study reveals possible hidden phenomena in the EU.

## Data Availability Statement

Publicly available datasets were analyzed in this study. This data can be found here: Eurostat. Eurostat database. Greenhouse gas emissions by source sector. http://appsso.eurostat.ec.europa.eu/nui/show.do?dataset=env_air_gge&lang=en. Global Burden of Disease Study. Results. 2019. Institute for Health Metrics and Evaluation: Seattle, United States. http://ghdx.healthdata.org/gbd-results-tool.

## Author Contributions

BG: conceptualization, investigation, writing—review and editing, visualization, supervision, project administration, and funding acquisition. MR: conceptualization, methodology, formal analysis, data curation, and writing—original draft preparation. VI: conceptualization, investigation, resources, writing—original draft preparation, writing—review and editing, and visualization. All authors contributed to the article and approved the submitted version.

## Funding

This research was supported by the Internal Grant Agency of FaME Tomas Bata University in Zlin: RO/2020/05; Economic quantification of marketing processes that focus on value increase for a patient in a process of system creation to measure and control efficiency in health facilities in the Czech Republic.

## Conflict of Interest

The authors declare that the research was conducted in the absence of any commercial or financial relationships that could be construed as a potential conflict of interest.

## Publisher's Note

All claims expressed in this article are solely those of the authors and do not necessarily represent those of their affiliated organizations, or those of the publisher, the editors and the reviewers. Any product that may be evaluated in this article, or claim that may be made by its manufacturer, is not guaranteed or endorsed by the publisher.
